# Emergence of non-susceptibility during persistent *Pseudomonas aeruginosa* bacteraemia in haematopoietic cell transplant recipients and haematological malignancy patients

**DOI:** 10.1093/jacamr/dlab125

**Published:** 2021-08-20

**Authors:** Lauren Fontana, Morgan Hakki

**Affiliations:** 1 Division of Infectious Diseases, University of Minnesota, Minneapolis, MN, USA; 2 Division of Infectious Diseases, Oregon Health and Science University, Portland, OR, USA

## Abstract

**Background:**

Systematic studies pertaining to the emergence of resistance during therapy of *Pseudomonas aeruginosa* bloodstream infections (BSIs) in haematopoietic cell transplant (HCT) recipients and haematological malignancy (HM) patients are lacking.

**Objectives:**

To determine how frequently non-susceptibility emerges during therapy of *P. aeruginosa* BSIs and to compare these findings with non-HCT/HM patients.

**Patients and methods:**

*P. aeruginosa* BSIs that occurred at our institution between 1 July 2012 and 31 October 2019 in HCT/HM patients and non-HCT/HM patients were identified. Episodes in which bacteraemia persisted while on appropriate therapy (‘persistent BSI’) were evaluated for emergence of non-susceptibility during therapy.

**Results:**

In total, 96 BSI episodes among 86 HCT/HM patients were analysed. Eight persistent BSI episodes (8.3%) occurred in eight patients (9.3%). Repeat susceptibility testing was performed in seven (87.5%) of these episodes. Non-susceptibility to the treatment agent emerged in five (71.4%) episodes and to any antipseudomonal agent in seven (100%) episodes. The 21 day mortality rate associated with persistent BSI was 87.5% (seven of eight), and it was 80% (four of five) among persistent BSI episodes in which non-susceptibility to the treatment agent emerged on therapy. Non-susceptibility to any antipseudomonal agent during persistent BSI emerged significantly more frequently in HCT/HM patients compared with non-HCT/HM patients.

**Conclusions:**

Non-susceptibility emerges frequently during persistent *P. aeruginosa* BSIs in HCT/HM patients, and this is associated with a high mortality rate. Our findings have implications for the management of persistent *P. aeruginosa* BSIs in these patients. Larger studies are needed to confirm and expand on our findings.

## Introduction

*Pseudomonas aeruginosa* is a frequent cause of bloodstream infection (BSI) among haematopoietic cell transplant (HCT) recipients and patients receiving chemotherapy for haematological malignancies (HM).^1–6^ In these patient populations, *P. aeruginosa* BSIs have been associated with crude mortality rates of up to 40%.^4^^,^^7–11^ A factor predictive of mortality during *P. aeruginosa* BSIs is infection with an MDR strain that may result in inappropriate empirical antibiotic therapy.^2^^,^^7^^,^^12^^,^^13^ While this highlights the importance of resistance as a determinant of outcome at the time of infection onset, the frequency during which resistance emerges during therapy is poorly understood.

Indeed, while the ability of *P. aeruginosa* to develop antimicrobial resistance during antibiotic exposure, both *in vitro and in vivo*, is well described,^14–20^ systematic studies pertaining to the emergence of resistance during therapy of *P. aeruginosa* BSIs in HCT/HM patients specifically, and all patients in general, are relatively lacking. A single, small study of HCT recipients documented emergence of non-susceptibility to any antibiotic during recurrent *P. aeruginosa* infection at any site in three of eight (37.5%) episodes.^9^ Among non-HCT/HM patients, studies examining serial cultures from various non-blood sites demonstrated that resistance emerged in anywhere from 5% to 30% of isolates.^16–19^^,^^21^^,^^22^ Most recently, emergence of resistance to any antipseudomonal agent among an isolate from any site—including colonization—within 30 days of treatment of *P. aeruginosa* BSI in hospitalized patients occurred in 12% of all episodes.^12^

The objective of this study was to determine how frequently non-susceptibility emerges during therapy of *P. aeruginosa* BSIs in HCT/HM patients. Additionally, to determine whether HCT/HM patients are unique with respect to this outcome, we compared our findings with non-HCT/HM patients with *P. aeruginosa* BSI.

## Patients and methods

We conducted a retrospective review of *P. aeruginosa* BSIs in all adult patients (age ≥18 years) at Oregon Health and Science University (OHSU) that occurred between 1 July 2012 and 31 October 2019. Cases were identified by reviewing blood culture data stored in OHSU’s infection control database. All demographic, microbiological and clinical information was extracted from the electronic medical record (EMR).

### Ethics

The study was approved by the OHSU Institutional Review Board.

### Antimicrobial susceptibility testing

Antimicrobial susceptibility testing on isolates from the first positive blood culture was performed as part of routine clinical care by the OHSU clinical microbiology laboratory using VITEK 2 (bioMérieux, Durham, NC, USA). Susceptibility testing on isolates from subsequent positive cultures was performed routinely at a 5 day interval from the first positive culture, or sooner if specifically requested. Results for cefepime, ceftazidime, meropenem, ciprofloxacin, piperacillin/tazobactam, gentamicin and tobramycin were reported as susceptible, resistant or intermediate, according to CLSI guidelines; laboratory reporting based on breakpoints for these antibiotics did not change during the study period.^23^^,^^24^ For the purpose of this study, a result reported as intermediate or resistant was considered ‘non-susceptible’.

### Definitions

A BSI episode was defined as the 14 day time period after the first blood culture that yielded *P. aeruginosa.*^25^ In the absence of an established definition, we defined ‘persistent BSI’ as a positive blood culture obtained after >48 h on appropriate antipseudomonal antibiotic therapy within an episode. As such, patients who died ≤48 h from onset of BSI were not included in the persistent BSI analysis. If a patient had a second positive blood culture for *P. aeruginosa* >14 days following the initial culture or completion of therapy for the initial infection, that was considered to be a second, independent episode and included as such in this study. Appropriate empirical antibiotic therapy was defined as receipt of at least one active agent based on susceptibilities.

Emergence of non-susceptibility during persistent BSI episodes was considered to have occurred if the strain isolated in the first blood culture was categorized as susceptible and a strain(s) isolated in subsequent blood cultures categorized as intermediate or resistant. Strains were categorized as MDR according to accepted criteria.^26^

Infections were considered hospital-associated if the BSI occurred ≥3 days following admission to, or ≤14 days following discharge from, OHSU.^27^ Neutropenia was defined as an absolute neutrophil count of <500 cells/μL at the time of first positive blood culture.^28^ Concomitant pneumonia was defined by the presence of new, focal radiographic consolidation(s) and symptoms (new onset cough, hypoxaemia), with or without microbiological documentation of *P. aeruginosa* in a respiratory tract sample, and absence of another defined aetiology during a BSI episode. The presence of a co-pathogen was defined as isolation of another bacteria or yeast from a blood culture drawn during the same *P. aeruginosa* BSI episode.

### Statistical analysis

Categorical variables were compared using the two-sided Fisher’s exact test.

## Results

### Patient characteristics and emergence of antimicrobial non-susceptibility during persistent BSI

There were 102 episodes of *P. aeruginosa* BSI among 92 HCT/HM patients (Table 1). In all cases, initial blood cultures were performed due to clinical suspicion of infection, as opposed to surveillance blood cultures in patients receiving high-dose steroids for graft-versus-host disease or another indication.

**Table 1.** Clinical characteristics at onset of *P. aeruginosa* BSI

Characteristic	*N* (%)^a^
Age, years, median (range)	61.5 (21–80)
Gender^b^	
male	60 (65.2)
female	32 (34.8)
Haematological malignancy	
acute myeloid leukaemia	45 (44.1)
acute lymphoblastic leukaemia	17 (16.7)
myelodysplastic syndrome	11 (10.8)
multiple myeloma	8 (7.8)
diffuse large B-cell lymphoma	3 (2.9)
Hodgkin’s lymphoma	3 (2.9)
chronic lymphocytic leukaemia	2 (2)
other^c^	13 (12.8)
Neutropenic	68 (66.7)
HCT recipient	50 (49)
CVC present at BSI	84 (82.3)
Hospital-associated infection	77 (75.5)
Antipseudomonal antibiotic exposure in previous 90 days^d^	82 (80.4)
fluoroquinolone	69 (67.6)
cefepime	45 (44.1)
piperacillin/tazobactam	18 (17.6)
carbapenem	13 (12.7)
Concomitant pneumonia	36 (35.3)
≥1 mg/kg/day prednisone (or equivalent)	19 (18.6)
Inappropriate empirical antibiotics	19 (18.6)
MDR *P. aeruginosa*	22 (21.6)
Co-pathogen^e^	19 (18.6)

a *N *=* *102 BSI episodes unless noted.

b Individual patients (*N *=* *92).

c Other lymphoma types (*n *=* *10), chronic myelocytic leukaemia (*n *=* *2), acute promyelocytic leukaemia (*n *=* *1).

d Some episodes were preceded by receipt of >1 class of antipseudomonal antibiotics.

e *Escherichia coli* (*n *=* *3), *Enterococcus faecalis* (*n *=* *1), *Candida* species (*n *=* *3), *Streptococcus* species (*n *=* *5), *Streptococcus pneumoniae* (*n *=* *1), *Stenotrophomonas maltophilia* (*n *=* *1), *Enterococcus faecium* (*n *=* *3), *Enterobacter cloacae* (*n *=* *1), MRSA (*n *=* *1).

Six patients died ≤48 h from BSI onset and therefore could not meet persistent BSI criteria by definition. Thus, the analysis of persistent BSIs was limited to 96 BSI episodes in 86 HCT/HM patients who survived >48 h after the initial blood culture. Eight BSI episodes in eight patients met criteria for a persistent BSI, representing 8.3% of qualifying BSI episodes and 9.3% of patients; no patient had more than one episode of persistent BSI. All patients with persistent BSI had initial blood cultures performed due to febrile neutropenia, and seven of the eight (87.5%) had repeat blood cultures performed due to clinical concern for ongoing infection including febrile neutropenia, septic shock and pneumonia; one had a follow-up blood culture performed to document clearance of BSI. No patient had duration of bacteraemia ≥14 days during a single episode and all episodes of persistent BSI occurred on the first episode of bacteraemia for each patient.

Characteristics of the eight HCT/HM patients with persistent BSI are provided in Table 2. All were neutropenic and had a central venous catheter (CVC) in place at the time of BSI onset. Notably, in six patients (75%) the CVC was removed within 48 h of infection onset; in the other two the CVCs were removed at 4 and 5 days following infection onset. Six (75%) had concomitant pneumonia. All eight patients had been exposed to an antipseudomonal antibiotic in the 90 days preceding the initial BSI. Seven patients (87.5%) were receiving an antipseudomonal antibiotic at the time of the initial BSI: a fluoroquinolone in six [levofloxacin (*n *=* *5) and ciprofloxacin (*n *=* *1)] and meropenem in one.

**Table 2.** Characteristics of HCT/HM patients with persistent BSI (*N *=* *8)

Characteristic	*N* (%)
Gender	
male	7 (87.5)
female	1 (12.5)
HCT recipient	2 (25)
Neutropenic	8 (100)
≥1 mg/kg/day prednisone (or equivalent)	3 (37.5)
Hospital associated infection	8 (100)
MDR strain	2 (25)
CVC in place	8 (100)
CVC removed within 48 h of BSI onset	6 (75)
Concomitant pneumonia	6 (75)
Antipseudomonal antibiotic exposure in previous 90 days^a^	8 (100)
levofloxacin	8 (100)
cefepime	3 (37.5)
meropenem	2 (25)
piperacillin/tazobactam	1 (12.5)
Antibiotic at time of BSI onset	7 (87.5)
fluoroquinolone	6 (85.7)
meropenem	1 (14.3)
Co-pathogen^b^	1 (12.5)

a Some episodes were preceded by receipt of >1 class of antipseudomonal antibiotics.

b *E.* faecalis BSI.

Susceptibility testing was repeated on at least one strain isolated in a blood culture subsequent to the initial culture in seven of eight (87.5%) episodes (Table 3). Emergence of non-susceptibility to any antipseudomonal antibiotic occurred in all 7 (100%). Non-susceptibility to the antibiotic being administered at the time of repeat susceptibility testing emerged in five of seven (71.4%): piperacillin/tazobactam in four (Patients 1, 2, 4 and 5) and cefepime in 1 (Patient 7). Emergence of non-susceptibility in these five episodes was documented after a median of 4 days of antibiotic exposure (range 2–12). Non-susceptibility to cefepime emerged in Patient 2 following discontinuation of 7 days of cefepime therapy and 3 days after initiating piperacillin/tazobactam. In the other two (Patients 3 and 6), non-susceptibility to an antibiotic in a different class than that being administered emerged. The antibiotics being administered at the time of each BSI (‘breakthrough antibiotic’) and the antibiotics used to treat each episode are provided (Table 3).

**Table 3.** Antimicrobial susceptibilities during persistent BSI in HCT/HM patients

Patient no.	Culture day	Susceptibilities^a^							Breakthrough antibiotic	Treatment	21 day mortality
		FEP	CAZ	CIP	GEN	MEM	TZP	TOB			
1											Y
isolate 1	0	S	S	R	S	I	S	S	LVX	FEP, TZP	
isolate 2	4	S	***R***	R	S	R	***R***	S	TZP	FEP	
isolate 3	7	S	***R***	R	S	R	***R***	S	FEP	TZP, TOB	
2											Y
isolate 1	0	S	S	R	S	S	S	S	LVX	FEP	
isolate 2	7	S	S	R	S	S	S	S	FEP	TZP	
isolate 3	10	***R***	***R***	R	S	***R***	***I***	S	TZP	MEM	
3											Y
isolate 1	0	S	S	S	S	S	S	S	none	MEM, TOB, TZP	
isolate 2	6	S	***I***	S	S	S	S	S	TZP	MEM	
4											N
isolate 1	0	S	S	R	I	R	S	S	LVX	FEP, TZP	
isolate 2	10	***I***	***R***	R	S	R	***R***	S	TZP	C/T, TOB	
5											Y
isolate 1	0	S	S	S	S	S	S	S	CIP	TZP	
isolate 2	2	***I***	***R***	S	S	S	***I***	S	TZP	TZP	
isolate 3	5	ND	ND	ND	ND	ND	ND	ND	TZP	IPM, TOB	
6											Y
isolate 1	0	S	S	R	S	I	S	S	LVX	TZP	
isolate 2	3	S	***I***	R	S	I	***I***	S	FEP	TOB, FEP	
isolate 3	5	ND	ND	ND	ND	ND	ND	ND	FEP	C/T	
7											Y
isolate 1	0	S	S	R	S	R	I	S	MEM	C/T, TOB, FEP	
isolate 2	12	***I***	***R***	R	S	R	I	***R***	FEP	FEP	
8											Y
isolate 1	0	S	S	R	R	S	S	I	LVX	FEP, TZP	
isolate 2	3	ND	ND	ND	ND	ND	ND	ND	TZP	TZP, AMK	

FEP, cefepime; CAZ, ceftazidime; CIP, ciprofloxacin; MEM, meropenem; TZP, piperacillin/tazobactam; TOB, tobramycin; C/T, ceftolozane/tazobactam; AMK, amikacin, IPM, imipenem; GEN, gentamicin; LVX, levofloxacin; S, susceptible; I, intermediate; R, resistant.

a Change from susceptible to non-susceptible categorization is shown in bold italics.

### Mortality associated with persistent BSI

Among all 102 BSI episodes in HCT/HM patients, the 21 day mortality rate was 27.4% (*n *=* *28). The 21 day mortality rate associated with persistent BSI was much higher at 87.5% (seven of eight) and was 80% (four of five) among persistent BSI episodes in which non-susceptibility to the treatment agent emerged on therapy (Table 3). The median time to death after the last positive culture in episodes of persistent BSI was 2 days (range 1–11). It should be noted that repeat susceptibility testing was incomplete in two patients who experienced 21 day mortality (Patients 6 and 8).

### Comparison of non-susceptibility emergence during persistent BSI between HCT/HM and non-HCT/HM patients

To determine whether the emergence of non-susceptibility during persistent *P. aeruginosa* BSI observed in HCT/HM patients differed from non-HCT/HM patients, persistent *P. aeruginosa* BSI episodes in these two groups were compared. Characteristics of non-HCT/HM patients and comparison to HCT/HM patients when applicable are provided in Table S1 (available as Supplementary data at *JAC-AMR* Online). As expected, significant differences in baseline characteristics existed between HCT/HM and non-HCT/HM patients as this was not intended to be a matched case-control comparison but rather an opportunity to highlight the fundamental differences between these two groups that may impact the emergence of non-susceptibility during persistent BSI.

Eight episodes of persistent BSI were identified in eight non-HCT/HM patients (Table S2). Compared with HCT/HM patients, the overall incidence of persistent BSI in non-HCT/HM patients (14%) was not significantly different (*P = *0.28) (Table 4). In non-HCT/HM patients, non-susceptibility emerged during two episodes (28.6%), in both cases to the treatment antibiotic (Table S3). Comparing the HCT/HM group to the non-HCT/HM group, non-susceptibility to any antipseudomonal antibiotic emerged significantly more frequently in HCT/HM patients compared with non-HCT/HM patients (Table 4). There was a numerical trend towards increased emergence of non-susceptibility to the treatment antibiotic among HCT/HM patients that did not reach statistical significance. Notably, no episode of persistent BSI in the non-HCT/HM population was associated with 21 day mortality.

**Table 4.** Emergence of non-susceptibility during persistent BSIs in HCT/HM and non-HCT/HM populations

Outcome	Population		*P* value
	HCT/HM, *N* (%)	non-HCT/HM, *N* (%)	
Persistent BSI: incidence^a^	8 (8.3)	8 (14.5)	0.28
Persistent BSI: emergence of non-susceptibility^b^			
to any antipseudomonal antibiotic	7 (100)	2 (28.6)	0.02
to the treatment antibiotic	5 (71.4)	2 (28.6)	0.28

a HCT/HM *N *=* *96, non-HCT/MH *N *=* *55.

b Limited to those with repeat susceptibilities available: HCT/HM *n *=* *7, non-HCT/HM *n *=* *7.

## Discussion

Despite the well-known ability of *P. aeruginosa* to develop resistance^29^ and the negative impact of infections with resistant strains in immunocompromised hosts including HCT recipients and HM patients,^7^ systematic studies pertaining to the emergence of resistance during therapy of *P. aeruginosa* BSIs in HCT/HM patients are lacking. We performed this study to determine how frequently this occurs during treatment of *P. aeruginosa* BSIs in these highly vulnerable patient populations. We found that persistent bacteraemia on therapy in HCT/HM patients is often accompanied by the emergence of non-susceptibility to the treatment agent and other antipseudomonal agents, and carries a high associated mortality rate, outcomes that appear to disproportionately impact HCT/HM patients compared with non-HCT/HM patients.

Among HCT/HM patients, non-susceptibility to at least one antipseudomonal antibiotic emerged in 100% of episodes of persistent BSI and to the treatment antibiotic in 71.4% of episodes. These rates are greater than in a previous small report with similar patients^9^ and when compared with the non-HCT/HM patients in this study and others.^12^^,^^16–19^^,^^21^^,^^22^ Additionally, non-susceptibility to the treatment agent emerged rapidly during persistent BSIs in HCT/HM patients, after a median of only 4 days of treatment. As our laboratory standard is to perform repeat testing on the fifth day of positive cultures, it is possible that non-susceptibility emerged sooner but was not recognized due to lack of susceptibility testing. In contrast, other studies not limited to HCT/HM populations and BSIs have generally indicated that more prolonged antibiotic exposure is required prior to the emergence of non-susceptibility *in vivo.*^12^^,^^15^^,^^19^^,^^21^

These findings may be related to several factors. First, our study was limited to BSIs, whereas other studies have included *P. aeruginosa* infections at all sites.^9^^,^^12^^,^^16–19^^,^^21^^,^^22^ Thus the site of infection may impact the emergence of non-susceptibility. However, even limited to BSIs, we found that non-susceptibility emerged more frequently in HCT/HM patients compared with non-HCT/HM patients. HCT/HM patients were more likely to have been exposed to antipseudomonal antibiotics prior to the onset of infection compared with non-HCT/HM patients (Table S2). This exposure, including levofloxacin for neutropenic prophylaxis and antipseudomonal β-lactams for febrile neutropenia, may prime resistance mechanisms,^29^ thereby lowering the threshold for phenotypic non-susceptibility to emerge during antipseudomonal antibiotic re-exposure. Differences in host factors such as neutropenia and the presence of a deep visceral nidus of infection such pneumonia may also contribute to the high incidence of non-susceptibility emergence during therapy in HCT/HM patients. Additional studies are required to define the underlying mechanisms and risk factors for the emergence of non-susceptibility during therapy of *P. aeruginosa* BSIs in HCT/HM patients.

We observed a 21 day mortality rate associated with all BSIs in HCT/HM patients of 27.4%, similar to previous reports in these patient populations.^1^^,^^2^^,^^6^^,^^8^^,^^9^^,^^11^ However, the mortality rates associated with persistent BSI episodes and with persistent BSI episodes in which non-susceptibility emerged while on therapy were much greater (87.5% and 80%, respectively). Additionally, the mortality rate associated with persistent BSI in HCT/HM patients was markedly greater than in non-HCT/HM patients (0%). Differences in underlying host characteristics may at least partially account for these differences, and the direct impact of the emergence of non-susceptibility on mortality during persistent BSI in HCT/HM patients could not be rigorously assessed due to the small number of episodes. Larger studies are required to address the impact of non-susceptibility emergence on outcomes in these patients.

Our findings have several clinically relevant implications for the management of persistent BSIs in HCT/HM patients. First, repeat susceptibility testing of isolates during persistent BSI, regardless of the duration of antibiotic exposure, appears to be warranted. Second, an empirical change in antibiotic therapy is indicated in the setting of persistent BSI. With the emergence of non-susceptibility not only to the treatment agent but also to agents in other classes, the use of an agent with more predictable activity against MDR *P. aeruginosa* such as ceftolozane/tazobactam^30–32^ and/or combination therapy may be appropriate depending on the local antibiogram and pending the results of repeat susceptibility testing.

Our study has several important limitations. The single centre nature of the work limits generalizability of our findings to similar patient populations at other centres. The retrospective design resulted in inconsistencies in obtaining follow-up blood cultures and performing repeat susceptibilities in the setting of persistent BSI. The relatively small number of episodes of persistent BSI prohibited an analysis of potential factors associated with the emergence of non-susceptibility and a direct analysis of the impact of non-susceptibility emergence on mortality. Due to lack of isolate availability, we were able to demonstrate genetic relatedness between the initial and subsequent strains during persistent BSI in only two episodes (data not shown), and therefore cannot definitely conclude that non-susceptibility during therapy emerged due to adaptation of the original strain versus selection of a different strain. However, previous studies have shown that *P. aeruginosa* strains during recurrent infection are most often genetically related to the strain during the initial episode.^9^^,^^25^^,^^27^ Regardless, the selection of unrelated resistant strains during therapy would not change the clinical implications of our findings in terms of the need for repeat susceptibility testing and empirical antibiotic changes.

In conclusion, we found that non-susceptibility during treatment emerges frequently and quickly during persistent *P. aeruginosa* BSIs in HCT/HM patients and is associated with a high mortality rate. Larger studies are needed to confirm and expand upon these findings.

## Funding

This study was conducted as part of routine work.

## Transparency declarations

None to declare.

## Supplementary data

Tables S1 to S3 are available as Supplementary data at *JAC-AMR* Online.

## Supplementary Material

dlab125_Supplementary_Data
